# Generalized Anxiety Disorder Among Physicians: A Cross-Sectional Study

**DOI:** 10.7759/cureus.74075

**Published:** 2024-11-20

**Authors:** Reda A Goweda, Abdurahman Hassan-Hussein

**Affiliations:** 1 Department of Community Medicine, Faculty of Medicine, Umm Al-Qura University, Makkah, SAU; 2 Department of Family Medicine, Faculty of Medicine, Suez Canal University, Ismailia, EGY

**Keywords:** anxiety, covid-19, gad-7, health care workers, physicians

## Abstract

Background: Generalized anxiety disorder (GAD) is defined as excessive worry that occurs on most days for at least six months, causes significant distress or impairment, and is associated with increased use of health services.

Methodology: A cross‑sectional study was carried out among physicians working in Saudi Arabia through an online questionnaire. The questionnaire consisted of a sociodemographic part and the validated Arabic version of the Generalized Anxiety Disorder‑7 (GAD‑7) questionnaire.

Results: The study included 110 physicians, 63 (57.3%) of whom were women with a mean age of 36.9 ± 10.0 years. Men represented 47 (42.7%) of the participants. The majority of the participants were non-Saudi (80, 72.7%). According to their scores on the GAD-7, 40 (36.4%) participants had no anxiety, 35 (31.8%) had mild anxiety, 25 (22.7%) had moderate anxiety, and 10 (9.1%) had severe anxiety. A score of 10 on the GAD-7 represented a reasonable cutoff point for identifying cases of anxiety. Accordingly, we found that 35 (31.8%) of the participating physicians had anxiety.

Conclusion: Physicians working in Saudi Arabia showed a significant rate of general anxiety disorder. Early detection, health education, and treatment are recommended.

## Introduction

Generalized anxiety disorder (GAD) and panic disorder (PD) are the most common anxiety disorders seen in primary healthcare among adults [1]. Patients with anxiety disorder are more likely than others to have chronic medical problems [2]. Integrating men­tal health services with primary care decreases treatment costs and increases access to care [3]. The Diagnostic and Statistical Manual of Mental Disorders, Fifth Edition (DSM-5) defines GAD as excessive worry that occurs on most days for at least six months and impairs quality of life, causing significant stress [4]. Excessive worry about small issues is commonly seen in patients with GAD in clinical practice. The disorder often manifests as somatic complaints as well as behavioral changes [5]. Peak onset is reported in many studies to be during late adolescence or early adulthood; however, GAD can also occur later in life [6]. There are several common factors associated with the disorder, such as sex, marital status, poor health, low educational achievement, and the presence of stressors. Etiology is multifactorial and includes psychological, social, biological, environmental, and genetic factors [7]. Females are twice as likely to have GAD than males [8], and healthcare workers, in particular, are among the high-risk groups, as they work in the frontline caring for people and facing heavy workloads, life-or-death decisions, and risk of infection. However, limited studies are available on anxiety among physicians. We aimed to screen for anxiety among physicians, assess severity, and identify risk factors that might increase susceptibility to GAD.

## Materials and methods

This was a cross-sectional study targeting physicians working in Saudi Arabia, carried out through an online questionnaire. The study included any graduated physician (men and women); medical students and interns were excluded. The sample size was calculated to be 110 participants, based on an estimated 10% prevalence rate of anxiety [9]. We followed a non-probability sampling technique (convenience sampling), as the sample was collected from easily accessible participants (online) with no randomization.

Data collection tool

The participating physicians were screened for GAD using a questionnaire, which was shared through a Google (Mountain View, CA) link in both English and Arabic. The questionnaire consisted of two sections: the first collected sociodemographic data, including age, sex, smoking, income, specialty, medical degree, marital status, children, and nationality. It also inquired about body mass index and diseases such as diabetes mellitus, hypertension, and other chronic and psychological diseases. The second section consisted of the Generalized Anxiety Disorder‑7 (GAD-7) questionnaire, a valid and efficient tool to screen for GAD and assess its severity in both clinical practice and research [10]. It is a seven-item, self-reported scale developed to test for the symptoms of GAD. The items assess some of the most salient diagnostic features of the disorder (feeling nervous, anxious, on edge, or excessively worried). Item responses, “Not at all,” “Several days,” “More than half the days,” and “Nearly every day,” are given the scores 0, 1, 2, and 3, respectively, which are added up to reveal the total score. Scores range from 0 to 21, with greater scores indicating severe GAD symptoms. The GAD-7 total score is interpreted as follows: below 5, normal; from 5 to 9, mild anxiety; from 10 to 14, moderate anxiety; and from 15 to 21, severe anxiety [11]. In this study, we accepted a score of 10 as a reasonable cutoff point for identifying cases of GAD.

Data analysis was carried out using IBM SPSS Statistics version 20.0 (IBM Corp., Armonk, NY). Mean and SD were estimated for quantitative variables. Frequencies and percentages were used for qualitative variables. Pearson's chi-square and Fisher exact tests were applied to observe associations between qualitative variables. A P-value of 0.05 or below was considered statistically significant.

The questionnaire began with a consent statement, ensuring participants’ agreement to partake in the study. The study was carried out after receiving ethical approval from the Umm Al-Qura University Deanship of Research (Approval Number: HAPO-02-K-012-2023-01-1386).

## Results

**Figure 1:**
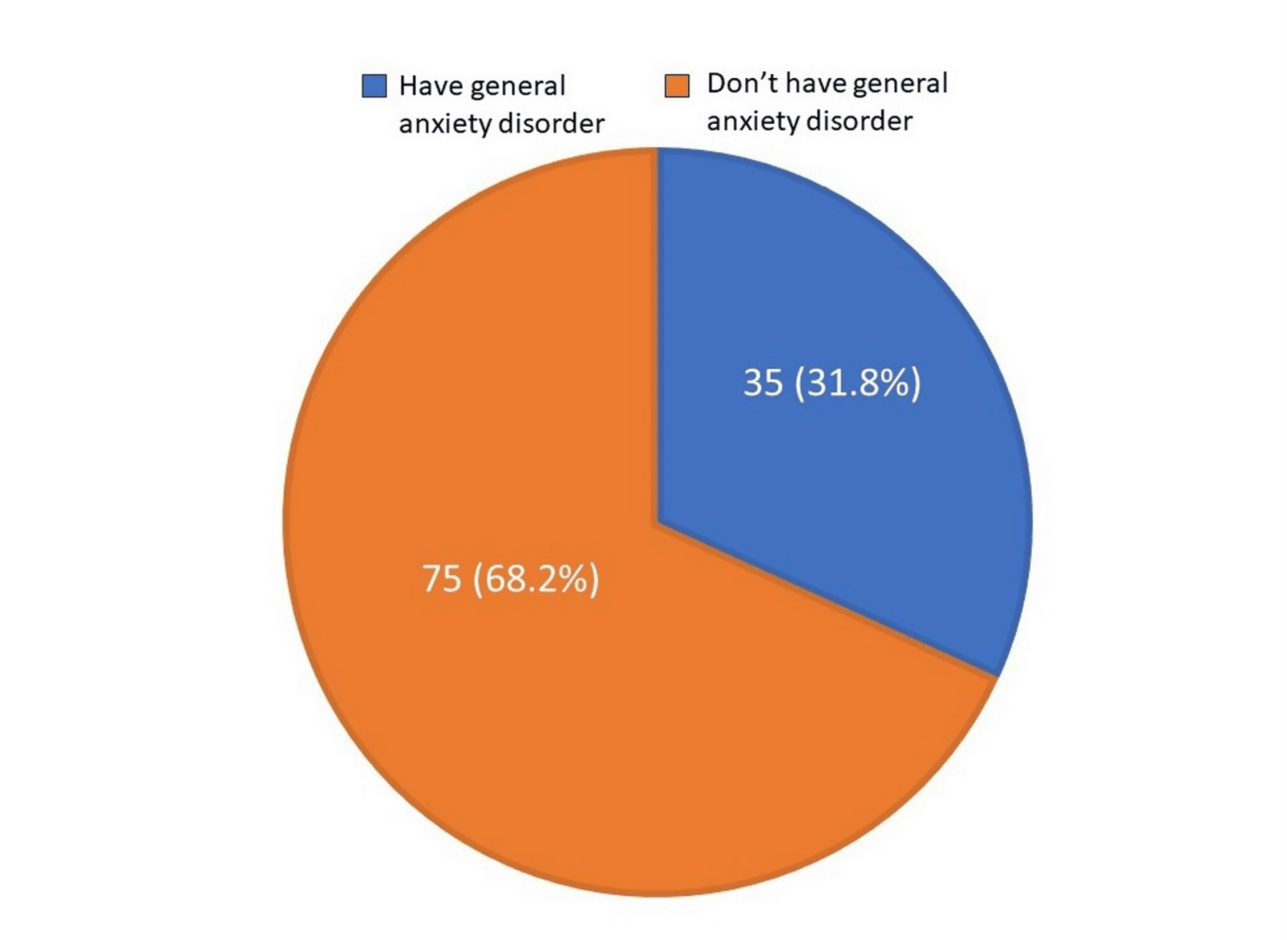
Prevalence of general anxiety disorder among the participating physicians (based on a score of 10 or higher on the General Anxiety Disorder-7 questionnaire).

A total of 110 participants responded to the questionnaire. Of the participants, 63 (57.3%) were women, and the mean participant age was 36.9 ± 10.0 years. Demographic data revealed that 81 (73.6%) of the participants were married, 89 (80.95%) had sufficient income, 80 (72.7%) were of non-Saudi nationality, seven (6.4%) were smokers, and 29 (26.4%) had chronic diseases. The majority were general practitioners, family physicians, or clinical pathologists (69, 62.7%) (Table 1). Based on a score of 10 or higher on the GAD-7, we found that 35 (31.8%) of the participating physicians had either moderate or severe GAD (Table 2 and Figure 1). We found no significant relationship between demographic variables and GAD (Table 3).

**Table 1:** General characteristics of the participants. ICU, intensive care unit; GP, general practitioner; BMI, body mass index.

		No. (%)
Sex	Male	47 (42.7%)
	Female	63 (57.3%)
Marital status	Married	81 (73.6%)
	Single	25 (22.7%)
	Divorced or widowed	4 (3.6%)
Children	Yes	78 (70.9%)
	No	32 (29.1%)
Income	Sufficient	89 (80.9%)
	Insufficient	21 (19.1%)
Nationality	Saudi	30 (27.3%)
	Non-Saudi	80 (72.7%)
Smoker	Yes	7 (6.4%)
	No	103 (93.6%)
Chronic diseases	Yes	29 (26.4%)
	No	81 (73.6%)
Specialty	Emergency and ICU	9 (8.2%)
	Internal medicine and pediatrics	20 (18.2%)
	Surgical (surgery, orthopedics, ophthalmology, ENT, etc.) or interventional	12 (10.9%)
	GP, family medicine, clinical pathology	69 (62.7%)
Medical degree	Resident	29 (26.4%)
	Specialist	49 (44.5%)
	Consultant	32 (29.1%)
Mean age (SD) (years)	36.9545 (10.06072)	
Mean BMI	28.348039 (5.040582)	

**Table 2:** Participants’ risk of general anxiety disorder according to GAD-7 scores. GAD-7, General Anxiety Disorder-7 questionnaire.

Score	No. (%)
Normal (<5)	40 (36.4%)
Mild anxiety (5-9)	35 (31.8%)
Moderate anxiety (10-14)	25 (22.7%)
Severe anxiety (15-21)	10 (9.1%)

**Table 3:** Relationship between general anxiety disorder and participant characteristics. GAD, general anxiety disorder; ICU, intensive care unit; GP, general practitioner; BMI, body mass index.

		GAD score ≥ 10, No. (%)		p
		Yes	No	
Sex	Male	11 (23.4%)	36 (76.6%)	0.076
	Female	24 (38.1%)	39 (61.9%)	
Marital status	Married	25 (30.9%)	56 (69.1%)	0.725
	Single	8 (32.0%)	17 (68.0%)	
	Divorced or widowed	2 (50.0%)	2 (50.0%)	
Children	Yes	23 (29.5%)	55 (70.5%)	0.274
	No	12 (37.5%)	20 (62.5%)	
Income	Sufficient	25 (28.1%)	64 (71.9%)	0.073
	Insufficient	10 (47.6%)	11 (52.4%)	
Nationality	Saudi	9 (30.0%)	21 (70.0%)	0.497
	Non-Saudi	26 (32.5%)	54 (67.5%)	
Smoker	Yes	1 (14.3%)	6 (85.7%)	0.284
	No	34 (33.0%)	69 (67.0%)	
Chronic diseases	Yes	9 (31.0%)	20 (69.0%)	0.556
	No	26 (32.1%)	55 (67.9%)	
Specialty	Emergency and ICU	3 (33.3%)	6 (66.7%)	0.104
	Internal medicine and pediatrics	8 (40.0%)	12 (60.0%)	
	Surgical (surgery, orthopedics, ophthalmology, ENT, etc.) or interventional	7 (58.3%)	5 (41.7%)	
	GP, family medicine, clinical pathology	17 (24.6%)	52 (75.4%)	
Medical degree	Resident	10 (34.5%)	19 (65.5%)	0.936
	Specialist	15 (30.6%)	34 (69.4%)	
	Consultant	10 (31.2%)	22 (68.8%)	
Age (years)		37.7143 ± 7.85745	36.6000 ± 10.96924	0.591
BMI		28.5531 ± 5.09750	28.2543 ± 5.04857	0.783

## Discussion

Healthcare workers are vulnerable to GAD, especially in view of the stressful work environment and lack of contact with families that they have endured since the beginning of the COVID-19 pandemic [12]. We found that 35 (31.8%) of the participating physicians had anxiety, which falls within the range of prevalence rates reported in the literature for anxiety among healthcare workers (13-55; 11.3%-50%).

In a study conducted in Jeddah, Saudi Arabia, one-fourth of the participating physicians (27.2%) had GAD, most of them at a moderate or severe degree [13]. A study from Ethiopia reported that healthcare workers experienced mild and moderate anxiety disorders (29.3% and 6.3%, respectively) [14]. The findings of a study in China revealed that 44.6% of healthcare workers exhibited symptoms of GAD [15].

Regarding severity, Elbay et al. reported that 16.3% of healthcare workers had mild, 13.1% had moderate, 10.6% had severe, and 11.5% had extremely severe anxiety symptoms [16]. Most of the physicians in the present study reported mild symptoms of anxiety; moderate and severe symptoms were less common among them, similar to the results of the Ethiopian study [14].

A cross-sectional study on GAD and its sociodemographic correlates among the general population in Saudi Arabia found that the prevalence of any degree of anxiety according to the GAD-7 was 62.1%, where 33.1% had mild, 15.7% had moderate, and 13.3% had severe anxiety [17]. Our results revealed a higher prevalence of GAD among female physicians but without statistical significance, which confirms the findings of the previously mentioned Saudi studies.

However, several studies from different countries also show similar results [9,18-20]. Females were found to have significantly higher levels of stress, affecting their mental health. Additionally, the prevalence and severity of anxiety in the present study were higher in physicians who were 38 years old or over (but without statistical significance). These results contrast with those reported by Maroufizadeh et al. [18] and Salari et al. [21] who found higher prevalence rates in people below 40 years old [22].

We found no significant relationship between participant BMI and GAD, similar to results reported by Aljurbua et al. [17]. In contrast to our results, Peltzer and Pengpid [23] and Yohannes et al. [24] found that chronic diseases have a negative effect on the prevalence of anxiety. We found no significant relationship between having chronic diseases and anxiety. To explain this, the physicians in our study may have a good understanding of their chronic conditions.

According to specialty, 17 (24.6%) of family physicians in our sample had GAD. In Pakistan, using the Aga Khan University Anxiety and Depression Scale, 39% of family practitioners were found to have anxiety disorders [25].

Acknowledging and addressing the inherent limitations of this study is essential. Firstly, there is a potential for selection bias as the study relied on voluntary participation, which may result in a non-representative sample of physicians. Therefore, caution should be exercised when generalizing the findings to the entire population of physicians in Saudi Arabia. Secondly, the use of self-reported data obtained through an online survey introduces the possibility of response bias. Participants might provide inaccurate or biased information, either knowingly or unknowingly. These limitations should be taken into account when interpreting the study's results and drawing conclusions. It is recommended that future research endeavors employ more diverse and representative samples to enhance the robustness of the findings.

## Conclusions

In conclusion, this research highlights the vulnerability of healthcare workers to GAD, particularly in the context of the COVID-19 pandemic. The prevalence of GAD among healthcare professionals is significant, emphasizing the need for targeted interventions and support systems to address anxiety and promote well-being. Further research is necessary to explore long-term impacts and develop effective prevention and management strategies.
